# Supplementary material for: Light in the tunnel or just a train; impact of supply chain finance solutions on financial service providers’ financial performance by mitigating financial risk
Source: PLoS One. 2023 Dec 13;18(12):e0292497. doi: 10.1371/journal.pone.0292497 (PMC10718461; doi:10.1371/journal.pone.0292497)
Supplement: S1 Annex — (DOCX) [file pone.0292497.s001.docx]

Annexure-I

| Sr. No | **SCFS** |
| --- | --- |
| 1 | Reverse Factoring |
| 2 | Accounts Receivables Financing |
| 3 | Purchase Order Financing |
| 4 | Agricultural Supply Chain Finance |
| 5 | Factoring |
| 6 | Online SCF Platform |
| 7 | Inventory Financing |
| 8 | Warehousing Financing |
| 9 | Buyer Direct Financing |
| 10 | Vendor-Managed Inventory |
| 11 | Raw Material Financing |
| 12 | Third Party Logistics Financing |
| 13 | Dynamic Discounting |
| 14 | Early Payment Discount Program |
| 15 | Buy Back Guarantee |
| 16 | Credit Guarantee |
| 17 | Bank Guarantee |
| 18 | Manufacturer Collateral |
| 19 | Supplier’s Subsidy |
| 20 | Pre-selling |
| 21 | Trade Credit |
