# Supplementary material for: Light in the tunnel or just a train; impact of supply chain finance solutions on financial service providers’ financial performance by mitigating financial risk
Source: PLoS One. 2023 Dec 13;18(12):e0292497. doi: 10.1371/journal.pone.0292497 (PMC10718461; doi:10.1371/journal.pone.0292497)
Supplement: Attachment — Submitted filename: Comments on PLOS ONE Paper.docx [file pone.0292497.s002.docx]

Comments on the paper “Light in the Tunnel or Just a train; Impact of Supply Chain Finance Solutions on Financial Service Providers' Financial Performance by Mitigating Financial Risk”

After reading the paper, I found that the author had a good research idea and research questions to explore. However, I found there are some issues with the execution of the research. I am listing my comments below:

1. The author used dummy variables to denote the Supply Chain Finance (SCF) solutions offerings by Financial Service Providers (FSPs). If the FSP offer a particular SCF solution in a given year, then it is coded as 1, else 0. OThusThus, reducing the SCF solutions offering to binary variables. However, I have a main concern of using Principal component Analysis (PCA) for data reduction or the creation of SCF index. PCA is suitable for reducing the numeric multivariate variables. If it is a mixed of numeric variables and binary variables, PCA can still be useful. There is no justification on the appropriateness of the PCA for reducing binary variables. Further, the paper makes no comment on the number of SCF solutions offered by banks. What was the maximum number of SCF solutions offered by FSPs? It is not clear out of how many binary variables (SCF solutions), the SCF Index was created.
2. *“Using principal component analysis (PCA), the Eviews index is created”-* The writing needs to be clearer. What is Eviews index? Do you mean SCF solution index is generated using PCA on Eviews software?
3. “*For example, if a bank offers account receivables finance or an early payment discount program, it is coded as 1 and 0 if not offered buyer direct financing*”. I have a concern with “0 if not buyer direct financing”. What does the author intend by this statement? Does the author mean that if the FSP is not offering any of the SCF solutions, then offering of buyer direct financing is assumed? If so then it could be wrong assumption. Just because a particular firm does not avail SCF solution from the FSP, does not mean it will always get buyer direct financing. A firm getting buyer direct financing may also simultaneously avail SCF solution from the bank.
4. Table 2a: The Adjusted R^2^ keeps on declining when new variables are added (Models 3 and 4). It means the main variables SCF solution index and SCF x Financial Risk are not contributing to the overall model. How do you reconcile with the overall results?

Table 2b and its interpretation: “*The coefficient is negative 0.0067, which means the moderating effect is interference rather than reinforcement. This suggests that the bank’s supply chain finance solution increases financial performance by increasing financial risk and supporting H2, which is the Supply chain finance solution that enables the financial performance of financial service providers to mitigate their financial risk”.* The author states that the coefficient is negative 0.0067 whereas Table 2b shows it to be positive. The interpretation “*This suggests that the bank’s supply chain finance solution increases financial performance by increasing financial risk and supporting H2, which is the Supply chain finance solution that enables the financial performance of financial service provider to mitigate their financial risk” is questionable.*

5. More care needs to be given in writing the overall manuscript.

Given the above issues, I feel the manuscript is not fit to be accepted at its current state. A lot of reworking will be required.
