# Supplementary material for: Light in the tunnel or just a train; impact of supply chain finance solutions on financial service providers’ financial performance by mitigating financial risk
Source: PLoS One. 2023 Dec 13;18(12):e0292497. doi: 10.1371/journal.pone.0292497 (PMC10718461; doi:10.1371/journal.pone.0292497)
Supplement: Attachment — Submitted filename: Manuscript Body_Camerinelli_review.docx [file pone.0292497.s003.docx]

**Title:** Light in the Tunnel or Just a train; Impact of Supply Chain Finance Solutions on Financial Service Providers' Financial Performance by Mitigating Financial Risk.

**Authors Detail:**

Munibah Munir^1#a*¶^

Nousheen Tariq Bhutta^1#a&^

1. Department of Management Sciences, Capital University of Science and Technology, Islamabad, Pakistan.

#a Current Address: Department of Management Sciences, Capital University of Science and Technology, Islamabad, Pakistan.

* Corresponding Authors

Email: [munibahmunirahmad@gmail.com](mailto:munibahmunirahmad@gmail.com) (MM)

¶ These authors contributed equally to this work

& These authors also contributed equally to this work

# **Abstract**

A newly emerging concept supply chain finance solutions is the atention grabber for financial service providers, buyers and suppliers. This study empirically analyzes the impact of supply chain finance solutions (SCFS), financial risk on financial service providers' financial performance using panel dataset of Asian Development Bank registered countries (Pakistan, China and Bangladesh) from 2012-2021. By breaking new ground, supply chain finance solution index is developed by combining several solutions to measure its impact on financial service provider financial performance. The results show a significant impact of supply chain finance solutions on financial performance of financial service providers. Furthermore, moderating role of financial risk shows reinforcement rather than interference on financial service provider financial performance. This research encourages financial service providers (banks) to embrace the supply chain finance solution to enhance financial performance and allows them to evaluate their supply chain finance solutions investments as a technique to mitigate financial risk.

**Key Words:** Supply Chain Finance Solutions, Financial Service Providers, Financial Performance, Financial Risk

# **Introdution:**

Supply chain finance is a newly emerging and innovative practice that has grabbed the attention of suppliers, buyers, and financial service providers (Zhao, 2015; Xu, 2018; Wuttke, 2019; Jia, 2020). Supply chain finance ensures efficient financial flow through the information and goods flow phases. Different research shows that it is a “win-win” situation for all participants (i.e., buyers, suppliers, and financial service providers) (Gelsomino, 2016; Lam, 2019; Ma, 2020). In any economy, the major contributors to the growth and survival of the banking sector are financial service providers and managing buyer-supplier relationships to reduce risk. Financial service providers, specifically banks, enhance their financial involvement in the global supply chain by offering different supply chain finance solutions according to the need of the supply chain participants (Martin and Hofmann, 2017).

Nevertheless, a financial service provider faces many risks that can destabilize their financial position, jeopardizing the stability of the whole economy and the financial performance of the financial service provider (Berger and Deyoung, 1997).

Most central bankers agreed that financial sector distress during massive financial crises was predominantly due to the bank's financial risk^1^, mostly conveyed by the bank's financial performance. Financial risk may not be avoided to enhance good financial performance; these two interdependent components must be evaluated simultaneously (Boermans, 2011).

Financial service providers are essential in all supply chain finance programs (Hofmann and Belin, 2011; Martin and Hofmann, 2017; Wuttke et al., 2019; Jia et al., 2020a). Financial service providers work to lower the risk for all supply chain participants who cannot repay the loan (Field and Meile, 2008; Pellegrino, 2019). That's why financial service providers are known as supply chain risk-takers and generate profits by performing this activity (Ma et al., 2020). Different studies show that supply chain finance initiatives allow financial service providers to have low-risk exposure by offering different but attractive discount rates (Fellenz, 2009; Martin and Hofmann, 2017; Song, 2018). Financial service providers’ financial risk may be influenced by supply chain initiatives (Lam, H. K., and Zhan, Y, 2021).

However, it is still unclear how the supply chain finance solutions index influences financial service providers’ financial risk. Some researchers suggested that financial services providers are evaluated by stakeholders based on their financial risk mitigation capability using supply chain finance solutions (Selviaridis and Norrman, 2014). However, there is very limited empirical evidence regarding the impact of supply chain finance solutions on their financial risk. Since Financial risk is one of the widely used market-based measurement ways and grabs the attention of many company investors and shareholders (Jo and Na, 2012; Tian and Xu et al., 2015). there is a need to figure out this research gap in the literature and whether supply chain solutions can help financial service providers mitigate financial risk.

Underpinned by a risk management perspective. Sitkin & Pablo (1992) suggested that an organization's internal control system with risk propensity leads toward risk level. Financial service providers take the risk contingent upon financial performance with the condition of financial risk propensity. Risk sharing reduces the quality risk and enhances performance levels (Tse, 2018). Financial service providers offer a supply chain finance solution contingent upon financial risk as it enhances performance. This study mainly focuses on the supply chain finance solution impact on the firm performance of financial service providers (banks) with the moderating role of financial risk. Different research has highlighted this research as having a high impact on the successful use of supply chain finance solutions (Lam, H. K., and Zhan, Y, 2021). For example, according to Lam, H. K., and Zhan (2021), supply chain financial initiatives play a critical role in the financial risk of financial service providers. Financial service providers’ financial risk reduces by initiating supply chain finance, but the impact of financial service providers’ financial performance remains empirically unexamined. As a result, other than investigating the direct effect of combined supply chain finance solutions on financial service providers’ financial performance, this study also examines the indirect moderating role of financial risk of financial service providers. Thus, this study attempts to answer the following question:

1. What is the impact of supply chain finance solutions on financial service provider’s financial performance?
2. What is the moderating role of financial risk on supply chain finance solutions and financial service providers’ financial performance?

To answer these questions, collect and use secondary panel data from multiple sources to open a new window with significant insights into the abovementioned relationships. The scarcity of empirical studies and the importance of supply chain finance solutions in the survival and economic situation make this paper an appealing substance of research. First, Supply chain finance is a new has grabbed the attention of suppliers, buyers, and financial service providers (Zhao et al., 2015; Xu et al., 2018; Wuttke et al., 2019; Jia et al., 2020); there is little research contribution that connects supply chain finance solutions impact with financial service providers finance performance. This research is the first to study the combined supply chain solutions relationship with financial service providers’ financial performance. By identifying the combined supply chain finance solutions relationship with financial service provider financial performance, this study gives future direction to scholars to further investigate the implications of financial performance on supply chain finance service users from buyers and suppliers as supply chain finance participants. Moreover, this study increases the knowledge of supply chain finance solutions by investigating the impact of financial risk on financial service providers' financial performance. Thus, the findings allow financial service providers to evaluate their offered supply chain finance solutions based on different firm resources.

## Theoretical Background

Bargaining power theory in which all parties are in an argumentative situation like contract writing, making agreements, or barraging contracts where one party has more influence over the other party. Kuhn, Lewin, and McNulty (1983), bargaining power is the relative ability of parties to exert influence over each other in a situation. In supply chain finance, bargaining is called bargaining if one party has strong power and exercises power to gain more over the weaker party (Crook and Combs, 2007). Financial service providers may not determine the risk level by offering supply chain finance solutions, but how financial risk affects their financial performance leads to a strong bargaining power exercise.

With more financial visibility for the financial service provider, they will gain more from the transaction over financial risk (Cho, Ke, J, and Han 2019). When banks offer supply chain finance solutions and act as risk-taker for the other two parties (buyers and suppliers) they can influence the other parties when they make the contract or negotiate the agreement. Banks with strong bargaining power towards other parties suggested that as risk-taker, they are more in the situation to extract influence over others to get offset against risk (Fabbri and Klapper, 2016). Bargaining power impact on financial service provider performance will be clearer via financial risk level, which forces the other parties to do what they will otherwise be less willing to do, for example, more growth (Lavie, Stettner, and Tushman 2010).

In this case, if all parties have equal bargaining rights, they perform perfect rights equally. In actual business or bank loan situation is different, and one party may have more power over the other. Banks hold longer cash and create a deficit for the other two parties that have to find finance solutions for different banks. In this case, banks have more bargaining power, and the other two parties have less access to credit, ultimately leading them to high costs (Oliveira, Kadapakkam, and Beyhaghi 2017). The weak bargaining power of parties has forced them to bear more risk and cost through the use of finance (Munson, Rosenblatt, M, and Rosenblatt 1999). In the case of financial service providers, banks put pressure on buyers and suppliers to accept late payments if they believe that other parties have resource slack. With this behavior, banks might be possible to increase their bargaining power as the other two parties prepare to formally file for bankruptcy (Fabbri and Klapper, 2016). As a financial service provider with strong bargaining power has an opportunity to have more favorable finance provider terms against other parties, a financial service provider may choose not to use power against a weak party due to concern about the party disruptions and overall will affect financial performance and growth (Wuttke et al., 2013).

# **Literature Review and Hypothesis Development:**

Supply chain management is collaborating and coordinating different supply chain parties' goods flow, information, and finance flow optimization (Mentzer et al., 2001). Different studies contribute to it as supply chain finance (SCF) can create a win-win situation for all the participants like suppliers, buyers, and financial service providers (Gelsomino et al., 2016), (Wang et al., 2020). Supply chain finance has a significant intersection of trade finance and the supply chain management field. Overall, all supply chain movement is to convert the material and information flow into the desired form along with the effective use of financial flows for all the parties in supply chain management (Hofmann et al., 2011). Supply chain finance’s basic idea is to deliver mutual value to all the supply chain parties (Cavenaghi, 2014). Selvaraj and Wesley (2020) added classic firm-oriented practices in supply chain finance now extended to deal with cash to cash conversion cycle, WACC *** spell acronym *** , cash flow management, and receivables and payables.

When measuring a firm financial performance and different financial decision phenomena, the Tobins q ratio is one of the indicators used in literature. Morck, Shleifer, and Vishny (1988) used Tobin's q ratio to explain cross-sectional returns implying a proxy for risk. Wernerfelt and Montgomery (1988) used Tobin's q to measure firm performance with the relative importance to measure share effect. In literature, the Tobins q ratio is not limited and is used to measure firm performance. All financial performance combines all the management factors that may be used for optimal profit achievement in any firm resources (Mansyur, 2017).

Bank's financial performance measurement, also known as profitability (Qin and Pastory, 2012) and Ruziqa (2013), measured financial performance by using return on assets (ROA) and net interest margin (NIM). Samad (2014) examined USA banks' failure factors and concluded return on assets has a significant performance factor for banks' failure.

***H1:*** Supply chain finance solutions have a positive impact on financial service providers' financial performance.

Common risks are liquidity, credit, market, and non-financial risks (Kassi, Rathnayake 2019). Out of all financial risks directly affect the company's financial position internally and in the market. Any fluctuation in return will lead to financial risk (Kioko et al., 2019). Due to financial movement in financial markets arises financial risk (Jorion and Khoury, 1996). Usually, it is linked with leverage and risk about all the due obligations and liabilities or not meeting with current assets. Due to the asymmetry of information among service providers, financial risk increases, and supply chain finance reduce this fact and reduce the uncertainty effect (Pfoh and Gomm, 2009; Shen, Choi, T, and Minner, 2019). Many service providers may fail to evaluate small businesses and their financial perspectives with conventional financing solutions (Moretto, Grassi, Caniato, Giorgino, and Ronchi 2019). Conversely, supply chain finance is offered by service providers and many substantial relevant business solutions. They maintained detailed transaction history and credit information for every supply chain participant (Xu et al., 2018).

Financial risk includes credit, liquidity, and operating risks, contributing to financial performance volatility (Dimitropoulos, 2010).

Sathyamoorthi and Mapharing (2020) examined that financial risk significantly impacts financial performance. Financial success is measured using ROA and ROE, net equity to total assets, gross debt to total assets, and loan-deposit ratio for financial risk management. For this analysis, the population was 10 Botswana commercial banks with secondary data set time 2011-2018 using different research tests (descriptive statistics, correlation, and regression analysis. Finding 11 of the study was interest rate had a significant negative effect on ROA and ROE.

Bolton et al. (2011) examined the financial risk relationship with ownership structure using Tobin's q ratio. They concluded that financial or leverage and business risk significantly positively impact inside ownership structure and firm financial performance.

***H2:*** Supply chain finance solutions enable financial service providers to mitigate their financial risk by improving their financial performance.

# Methodology

In this research, Asian Development bank registered countries, Pakistan, China and Bangladesh 25 banks data used as sample from 2012-2021. This research sample covers conventional banks and excludes Islamic and saving investment banks due to their different business models. It focuses on the country level rather than the firm-level and individual SCF solutions level, such as reverse factoring and inventory financing. All variables’ data are collected on an annual base from the bank's official websites and annual reports of banks.

## **Variables Description:**

### **Financial Performance**

Financial performance is measured in two ways; internal and external. Internal financial performance is estimated using the ROA of the year. First, ROA is computed as the net income ratio to total assets. (Said and Tumin, 2011). The value has been transformed into a log of ROA to improve the data normality. The ROA is selected because the return on assets shows the management's ability to make a profit from the bank’s assets

$$ROA=\frac{PAT}{TA} \times100$$

ROA is the return on assets, PAT is profit after tax, and TA is total assets.

Second, the external market is measured by Tobin’s Q. this ratio is consistent with the Malikel and fama (1970) established efficient market hypothesis. This ratio is used to measure the company's potential future growth and existing assets. Tobin's Q ratio measures the investor's future expectations with the business's current strategies for evaluation (Christensen et al., 2010; Demsetz & Villalonga, 2001; Ehikioya, 2009; Rodriguez Fernandez, 2016).

$$Q ratio= \frac{Total market value of firm}{Total Assets Value}$$

Where the Q ratio is Tobin's Q, the company's total market value is outstanding stock and debt, and the value of the total assets is the replacement cost of the company's assets (book value) (Christensen et al., 2010).

### **Supply chain Finance solution Index**

Supply Chain Finance solutions: After identifying supply chain finance solutions offered by the bank's website and annual report, we coded the offered supply chain finance solutions as 1 and 0 for not offered solution. For example, if a bank offers account receivables finance or an early payment discount program, it is coded as 1 and 0 if not offered buyer direct financing. Using principal component analysis (PCA), the E-views index is created. The principal component analysis is a way for index creation for a group of variables with similar information. PCA allows having maximum information without those variables which have multicollinearity. As supply chain finance solutions list some variables that are similar in the information that can cause multicollinearity, created an index by using PCA.

### **Financial Risk**

Financial risk is the probability of losing profit based on the bank's financial characteristics (Peng, 2011). Financial risk is the average of two risks, credit risk and liquidity risk, contributing to financial performance (Dimitropoulos et al., 2010). Credit risk is measured as capital adequacy ratio (CAR) and non-performing loans (NPL). At the same time, the liquidity ratio is measured as total loans divided by total deposits.

$$FR=\frac{CR+LR}{2}$$

Where FR is financial risk, CR is credit risk, and LR is liquidity risk.

Control Variables: The research model includes bank-specific control variables. The bank-specific variables are tier 1 capital ratio (T1), loan-to-asset ratio (LA), other earnings assets (OEA), and bank size (BS). T1 is used to control for differences in banking sector development, while the other variables (LA, OEA, and BS) are used to control for systemic, idiosyncratic, and market risks of the bank (Han et al., 2017; Hsu et al., 2016; Lam, 2018; Luo and Bhattacharya, 2009)

## **Empirical Model**

In this research, a panel data analysis is used. To measure the impact of the supply chain finance solutions index and financial risk on the financial service provider's finance performance as the following form in

${FP}_{it}= \beta_{o}+{\beta_{1}SCFSI}_{it}{{+ \beta}_{2}FR}_{it}+{\beta_{3}SCFSI}_{it}{\times FR}_{it}{{+\beta}_{4}BS}_{ij}{{+ \beta}_{5}LA}_{it}{{+ \beta}_{6}TCA}_{it}{{+ \beta}_{7}EA}_{it}+\alpha_{i}+ \delta_{t}+\varepsilon_{it}$	*(1)*

Where i and t are banks and year indices, respectively; SCFSI is supply chain finance solutions index, FR is financial risk, BS is bank size, LA is leverage, TCA is tier 1 capital ratio, EA is earning assets, a_i_ = firm-level effect δ_t_ = year-level effect and ε = error term

regression investigation in this study will focus on two main approaches: fixed and random effects models.

## **Fixed Effect Regression Model**

This research objective was to analyze the direct impact of the supply chain finance solution index on the financial performance of financial service providers (H1) and moderating role of financial risk (H2). In the analysis, there were many challenges. First, although by adding many control variables like bank size, earning assets, tier-1 capital ratio, and loan-to-assets ratio, might ignore some firm unobservable characteristics related to the firm’s decision to offer supply chain finance solutions and simultaneously its financial performance, raising possible concerns of endogeneity (Lu et al., 2018). Second, this research sample time covered 10 years from 2012 to 2021, some unobservable time-specific effects (COVID-19) might also explain a firm's financial performance over time. Lastly, although we have hypothesized the impact of firm supply chain finance solutions on its financial performance, firms' financial performance might also affect the offer of supply chain finance solutions, raising possible reverse causality issues (Wooldridge, 2010). To address these challenges, using different ways. First, all sample firms provided supply chain finance solutions between 2012-2021, with this firm-level fixed-effect estimation possible (Bockstedt et al., 2015; Shan and Zhu, 2013).

Furthermore, this research was interested in how firms' supply chain finance solutions affect their financial performance over time. With this approach, the firm-level-fixed-effect helped to remove any unobservable firm characteristics time-variant like corporate culture and decision-making that may be related to offering the firm's supply chain finance solutions and financial performance of financial service providers; this will lead to reduced endogeneity concern and make within-firm consistent. Furthermore, this research included a year-level fixed effect estimation to remove any unobservable specific time effect that any economic trends or events can cause. Add a one-year lag to the dependent variable (financial performance, as t+1) and all independent variables like supply chain finance solutions index and financial risk to address the reverse causality concern. The fixed effect regression equation is:

${ROA}_{it}= \beta_{o}+{\beta_{1}SCFSI}_{it}{{+ \beta}_{2}FR}_{it}+{\beta_{3}SCFSI}_{it}{\times FR}_{it}{{+\beta}_{4}BS}_{ij}{{+ \beta}_{5}LA}_{it}{{+ \beta}_{6}TCA}_{it}{{+ \beta}_{7}EA}_{it}+\alpha_{i}+ \delta_{t}+\varepsilon_{it}$	*(2)*

${TOQ}_{it}= \beta_{o}+{\beta_{1}SCFSI}_{it}{{+ \beta}_{2}FR}_{it}+{\beta_{3}SCFSI}_{it}{\times FR}_{it}{{+\beta}_{4}BS}_{ij}{{+ \beta}_{5}LA}_{it}{{+ \beta}_{6}TCA}_{it}{{+ \beta}_{7}EA}_{it}+\alpha_{i}+ \delta_{t}+\varepsilon_{it}$	*(3)*

Where i and t are banks and year indices, respectively; a_i_ is a firm-level effect, δ_t_ is the year-level effect, and ε is an error term. $\beta1$ supply chain solutions index on financial service providers' financial performance (H1) and $\beta3$the financial risk moderating role (H2)

The first is the F-test to determine whether a mixed regression or fixed effect model should be used. The F-test p-value is 0.0000, which is significant and uses a fixed-effect model. Hausmann test p-value is 0.0000 rejecting the null hypothesis and using the fixed-effect model over the random-effect model.

# **Results:**

Table 1 shows the correlation, mean and standard deviations of all variables. Fixed-effect model regression results are shown in Tables 2 a and b. There are 4 regression models in table-2a and 2b: Model-1 shows the results of all control variables as well as banks and year-level fixed effects; Model-2 shows the direct effect of the financial risk, Model-3 shows the direct effect of the supply chain finance solutions index and Model-4 shows the moderating role of financial risk. All three models are significant statistically (p<0.01) and based on F-statistics, with R-squared value from 0.54 to 0.98. Observations for each bank for the three models is 210, which means there are around 10 years of observations for each sample bank (unbalanced panel).

| **Table 1. Correlation Matrix** | | | | | | | | |
| --- | --- | --- | --- | --- | --- | --- | --- | --- |
|  | **1** | **2** | **3** | **4** | **5** | **6** | **7** | **8** |
| **ROA** | 1 |  |  |  |  |  |  |  |
| **TOQ** | -0.2484 | 1 |  |  |  |  |  |  |
| **FR** | 0.2285 | -0.0044 | 1 |  |  |  |  |  |
| **SCFSI** | -0.1616 | 0.1971 | -0.154 | 1 |  |  |  |  |
| **BS** | -0.2725 | -0.1241 | 0.146 | -0.1722 | 1 |  |  |  |
| **EA** | 0.3205 | -0.1431 | -0.007 | 0.0670 | -0.6246 | 1 |  |  |
| **LA** | -0.4008 | -0.1259 | -0.157 | -0.1439 | 0.1275 | -0.336 | 1 |  |
| **TCA** | 0.0499 | -0.0112 | 0.173 | -0.1495 | 0.1778 | -0.015 | -0.1232 | 1 |
| **Mean** | 0.0494 | -1.6861 | -1.1472 | 0.4171 | -0.3784 | 2.6197 | -0.8785 | -1.9238 |
| **Standard Deviation** | 0.0676 | 1.9846 | 0.6128 | 0.8506 | 0.7892 | 0.0715 | 0.3948 | 0.5329 |

| **Table 2a, Fixed effect test results of Return on Assets** | | | | |
| --- | --- | --- | --- | --- |
| **Variables** | **Model 1** | **Model 2** | **Model 3** | **Model 4** |
| **Bank Size** | 1.2027 | 1.3994 | 1.6478 | 1.7313 |
|  | (0.3913) | (0.3608) | (0.4282) | (0.4587) |
| **Loan to Asset** | -0.1303 | -0.1034** | -3.4643 | -3.8275 |
|  | (0.3405) | (-0.8383) | (0.2504) | (0.2033) |
| **Tier 1 capita** | 0.90214** | 0.7466 | 0.8825* | 0.8387* |
|  | (0.000) | (7.6918) | (0.0732) | (0.0946) |
| **Earning Assets** | 0.4638** | 0.1019 | 0.4192* | 0.4325 |
|  | (0.000) | (0.000) | (0.0947) | (0.1414) |
| **Financial Risk** |  | 0.4188** | 0.5694** | 0.0033 |
|  |  | (0.000) | (0.0288) | (0.9945) |
| **Supply Chain Finance Solution Index** |  |  | -0.0655 | 0.9888* |
|  |  |  | (0.6372) | (0.078) |
| **Supply Chain Finance Solution Index × Financial Risk** |  |  |  | 1.0209  (0.1092) |
| **Intercept** | -4.8821 | -5.2576 | -6.508 | -7.6466 |
|  | 0.173 | 0.1782 | 0.3104 | 0.2203 |
| **Firm-level fixed effects** | Yes | Yes | Yes | Yes |
| **Year-level fixed effects** | Yes | Yes | Yes | Yes |
| **Number of observations** | 210 | 210 | 210 | 210 |
| **F-statistic** | 21.7109 | 23.4759 | 4.5317 | 4.5379 |
| **Prob(F-statistic)** | 0.000 | 0.000 | 0.000 | 0.000 |
| **Adjusted R^2^** | 0.7659 | 0.7852 | 0.5446 | 0.5566 |
| Note: 10 % (*) and 5% (**) | | | |  |

| **Table 2b, Fixed effect test results of Tobin’s Q** | | | | |
| --- | --- | --- | --- | --- |
| **Variables** | **Model 1** | **Model 2** | **Model 3** | **Model 4** |
| **Bank Size** | 0.0034 | 0.0037 | 0.0127 | 0.0133 |
|  | (0.6844) | (0.6472) | (0.2851) | (0.2064) |
| **Loan to Asset** | -0.0002 | -0.0002 | -3.4643 | -0.0337 |
|  | (0.7806) | (0.8291) | (0.2504) | (0.3468) |
| **Tier 1 capita** | 0.0003 | -4.2507 | -0.0037 | -0.0040 |
|  | (0.8044) | (0.9997) | (0.3672) | (0.3486) |
| **Earning Assets** | 0.0043** | 0.0044** | 0.0032 | 0.0032 |
|  | (0.0000) | (0.0000) | (0.3665) | (0.3559) |
| **Financial Risk** |  | 0.0007* | 0.0011* | -0.0026** |
|  |  | (0.0656) | (0.007) | (0.026) |
| **Supply Chain Finance Solution Index** |  |  | 0.0037 | 0.0106** |
|  |  |  | (0.1401) | (0.0000) |
| **Supply Chain Finance Solution Index ×**  **Financial Risk** |  |  |  | 0.0067***  (0.0029) |
| **Intercept** | 0.0118 | 0.0111 | -0.0175 | -0.0250 |
|  | (0.571) | (0.5857) | (0.7843) | (0.6924) |
| **Firm-level fixed effects** | Yes | Yes | Yes | Yes |
| **Year-level fixed effects** | Yes | Yes | Yes | Yes |
| **Number of observations** | 210 | 210 | 210 | 210 |
| **F-statistic** | 318.3026 | 309.526 | 164.7493 | 161.2075 |
| **Prob(F-statistic)** | 0.0000 | 0.0000 | 0.0000 | 0.0000 |
| **Adjusted R^2^** | 0.9804 | 0.9805 | 0.9823 | 0.9827 |
| Note: 10 % (*), 5% (**) and 1% (***) | | | |  |

Table 2a shows the results of the return of assets effect on supply chain finance solutions is significant (p<0.10); in model 4, its coefficient is 0.9888, and with Tobin's Q, its coefficient is 0.0106 means it has a positive relation with financial service providers financial performance. This means that a 1% supply chain finance solution increase will increase financial performance by 0.9888 and 0.0106, respectively. Thus, H1 is accepted based on model number 4.

Financial risk as a moderating effect, the interaction between supply chain finance solutions and financial risk is significant (p<0.01), as shown in model 4 table 2b. The coefficient is negative 0.0067, which means the moderating effect is interference rather than reinforcement. This suggests that the bank’s supply chain finance solution increase financial performance by increasing financial risk and supporting H2, which is the Supply chain finance solution that enables the financial performance of financial service provider to mitigate their financial risk.

# **Conclusion**

This research reveals the effect of supply chain finance solutions on financial service providers' financial performance. First, it demonstrates that supply chain finance solutions enhance the financial performance of financial service providers. Moreover, financial service providers' financial performance financial risk mitigates with supply chain finance solutions. As per Hofmann and Belin (2011), supply chain finance is a capital strategy that can use for financing terms to relax and increase the financial performance of financial service providers. Following the literature pattern, with the help of supply chain finance solutions, financial service providers' financial risk can mitigate and enhance financial performance because financial risk impacts financial service providers and overall stakeholders (Aggarwal et al., 2011). It has to consider for supply chain finance solutions development and implementation. Therefore, the research findings indicate that financial service providers can use supply chain finance solutions as a strategic move to enhance financial performance and mitigate their financial risk, as shown in Tables 2 a and b (model 4).

## **Research Implications**

This research has some implications for future research. First, some researchers have highlighted that supply chain finance solutions can provide a “win-win” situation (Gelsomino et al., 2016; Lam et al., 2019; Ma et al., 2020), the financial risk implication of supply chain finance solutions and its impact of financial service providers financial performance are not clear. This is one of the studies to offer empirical evidence on how the supply chain finance solution index affects financial service providers' financial performance with financial risk. Scholars in finance allow them to extend the finance literature boundaries and investigate supply chain finance solutions associations with non-financial strategies linked with financial risk.

# **References**

1. Agrawal, N., Dai, Q., and Walden, E.A., 2011. The more, the merrier .How the number of partners in a standard-setting initiative affects shareholder's risk and return. MIS Quarterly, 35(2), 445-462.
2. Berger, Allen N., Deyoung, Robert, 1997. Problem loans and cost efficiency in commercial banks board of governors of the federal reserve system. J. Bank. Finance 21 (6), 849–870.
3. Boermans, M.A. (2011). Firm Performance under Financial Constraints and Risks: Recent Evidence from Micro Finance Clients In Tanzania, HU University of Applied Sciences Utrecht.
4. Bolton, P., Chen, H., and Wang, N. (2011). A unified theory of Tobin’s q, corporate investment, financing, and risk management. The Journal of Finance, 66:1545–1578.
5. Cho, W., Ke, J.-y. F., and Han, C. (2019). An empirical examination of the use of bargaining power and its impacts on supply chain financial performance. Journal of Purchasing and Supply Management, 25:100550.
6. Cavenaghi, E. (2014). Supply-chain finance: The new frontier in the world of payments. Journal of Payments Strategy & Systems, 7:290–293.
7. Christensen, J., Kent, P., & Stewart, J. (2010). Corporate governance and company performance in Australia. Australian Accounting Review, 20(4), 372–386.
8. Crook, T. R., and Combs, J. G. (2007). Sources and consequences of bargaining power in supply chains. Journal of operations management, 25:546–555.
9. Demsetz, H., & Villalonga, B. (2001). Ownership structure and corporate performance. Journal of Corporate Finance, 7(3), 209–233.
10. Dimitropoulos, P. E., Asteriou, D., and Koumanakos, E. (2010). The relevance of earnings and cash flows in a heavily regulated industry: Evidence from the greek banking sector. Advances in Accounting, 26:290–303.
11. Ehikioya, B. I. (2009). Corporate governance structure and firm performance in developing economies: Evidence from Nigeria. The International Journal of Business in Society, 9(3), 231–243.
12. Fabbri, D. and Klapper, L. F. (2016). Bargaining power and trade credit. Journal of corporate finance, 41:66–80.
13. Fellenze, M.R., Augustenborg, C., Brady, M., and Greene, J., 2009. Requirements for an evolving model of supply chain finance: A technology and service providers perspective. Communications of the IBIMA, 10(29), 227-235.
14. Gelsomino, L. M., Mangiaracina, R., Perego, A., and Tumino, A. (2016). Supply chain finance: a literature review. International Journal of Physical Distribution & Logistics Management, 46:2014–0173.
15. Han, K., Mittal, V., and Zhang, Y., 2017. Relative strategic emphasis and firm-idiosyncratic risk: The moderating role of relative performance and demand instability. Journal of Marketing, 81(4), 25-44.
16. Hofmann, E., Belin, O., et al. (2011). Supply chain finance solutions. Springer.
17. Hsu, L., Fournier, S., and Srinivasan, S., 2016. Brand architecture strategy and firm value: How leveraging, separating, and distancing the corporate brand affects risk and returns. Journal of the Academy of Marketing Science, 44(2), 261-280.
18. Jia, F., Blome, C., Sun, H., Yang, Y., and Zhi, B. (2020). Towards an integrated conceptual framework of supply chain finance: An information processing perspective. International Journal of Production Economics, 219:18–30.
19. Jo, H., and Na, H., 2012. Does CSR reduce firm risk? Evidence from controversial industry sectors. Journal of Business Ethics, 110(4), 441-456.
20. Jorion, P. and Khoury, S. (1996). Financial risk management. Cambridge/Massachusetts
21. Kassi, D. F., Rathnayake, D. N., Louembe, P. A., and Ding, N. (2019). Market risk and financial performance of non-financial companies listed on the Moroccan stock exchange. Risks, 7:20.
22. Kioko, C., Olweny, T., and Ochieng, L. (2019). Effect of financial risk on the financial performance of commercial banks in Kenya listed on the Nairobi stock exchange. The Strategic Journal of Business & Change Management, 6:1936–1952.
23. Kuhn, J. W., Lewin, D., and McNulty, P. J. (1983). Neil w. chamberlain: a retrospective analysis of his scholarly work and influence. British journal of industrial relations, 21:143–160.
24. Lam, H.K., 2018. Doing good across organizational boundaries: Sustainable supply chain practices and firms’ financial risk. International Journal of Operations & Production Management, 38(12), 2389-2412.
25. Lam, H. K., and Zhan, Y. (2021). The impacts of supply chain finance initiatives on firm risk: evidence from service providers listed in us. International Journal of Operations & Production Management, 41:383 – 409.
26. Lavie, D., Stettner, U., and Tushman, M. L. (2010). Exploration and exploitation within and across organizations. Academy of Management Annals, 4:109–155
27. Luo, X., and Bhattacharya, C.B., 2009. The debate over doing good: Corporate social performance, strategic marketing levers, and firm-idiosyncratic risk. Journal of Marketing, 73(6), 198-213.
28. Ma, H.-L., Wang, Z., and Chan, F. T. (2020). How important is supply chain collaborative factors in supply chain finance? a view of financial service providers in china. International Journal of Production Economics, 219:341–346
29. Malkiel, B. G., & Fama, E. F. (1970). Efficient capital markets: A review of theory and empirical work. The Journal of Finance, 25(2), 383–417.
30. Mansyur, N. (2017). Impact financial risk on financial performance bank in Indonesia. The International Journal Of Business & Management, 5:305–31
31. Martin, J. and Hofmann, E. (2017). Involving financial service providers in supply chain finance practices: Company needs and service requirements. Journal of Applied Accounting Research.
32. Mentzer, J. T., DeWitt, W., Keebler, J. S., Min, S., Nix, N. W., Smith, C. D., and Zacharia, Z. G. (2001). Defining supply chain management. Journal of Business Logistics, 22:1–25.
33. Morck, R., Shleifer, A., and Vishny, R. W. (1988). Management ownership and market valuation: An empirical analysis. Journal of financial economics, 20:293–315.
34. Moretto, A., Grassi, L., Caniato, F., Giorgino, M., and Ronchi, S. (2019). Supply chain finance: From traditional to supply chain credit rating. Journal of Purchasing and Supply Management, 25:197–217.
35. Munson, C. L., Rosenblatt, M. J., and Rosenblatt, Z. (1999). The use and abuse of power in supply chains. Business Horizons, 42:55–56.
36. Oliveira, M., Kadapakkam, P.-R., and Beyhaghi, M. (2017). Effects of customer financial distress on supplier capital structure. Journal of Corporate Finance, 42:131–149.
37. Peng, Y. et al., (2011). An Empirical Study of Classification Algorithm Evaluation for Financial Risk Prediction, Applied Soft Computing, 11, pp. 2906-2915.
38. Pfohl, H.-C. and Gomm, M. (2009). Supply chain finance: optimizing financial flows in supply chains. Logistics Research, 1:149–161.
39. Qin, X. and Pastory, D. (2012). Commercial banks profitability position: The case of Tanzania. International Journal of Business and Management.
40. Rodriguez-Fernandez, M. (2016). Social responsibility and financial performance: The role of good corporate governance. BRQ Business Research Quarterly, 19(2), 137–151.
41. Ruziqa, A. (2013). The impact of credit and liquidity risk on bank financial performance: the case of Indonesian conventional bank with total assets above 10 trillion rupiahs. International Journal of Economic Policy in Emerging Economies, 6:93–106.
42. Said, R.M. & Tumin, M.H. (2011). Performance and Financial Ratios of Commercial Banks in Malaysia and China, International Review of Business Research Papers, 7 (2), pp. 157-169.
43. Sathyamoorthi, C., Mapharing, M., Mphoeng, M., Dzimiri, M., et al. (2020). Impact of financial risk management practices on financial performance: Evidence from commercial banks in Botswana. Applied Finance and Accounting, 6:25–39.
44. Selvaraj, J. J. A. and Wesley, J. R. (2020). Integrated supply chain risk management capabilities and its impact on supply chain demand management-an empirical study. International Journal of Business Information Systems, 34:447–462.
45. Shen, B., Choi, T.-M., and Minner, S. (2019). A review on supply chain contracting with information considerations: information updating and information asymmetry. International Journal of Production Research, 57:4898–4936.
46. Sitkin, S.B., and Pablo, A.L., 1992. Reconceptualizing the determinants of risk behavior. Academy of Management Review, 17(1), 9-38.
47. Song, H., Yu, K., and Lu, Q., 2018. Financial service providers and banks’ role in helping SMEs to access finance. International Journal of Physical Distribution & Logistics Management, 48(1), 69-92.
48. Tian, F., and Xu, S.X., 2015. How Do Enterprise Resource Planning Systems Affect Firm Risk? Post-Implementation Impact. MIS Quarterly, 39(1), 39-60.
49. Tse, Y.K., Zhang, M., and Jia, F., 2018. The effects of risk and reward sharing on quality performance. International Journal of Operations & Production Management, 38(12), 2367-2388.
50. Wang, Z., Wang, Q., Lai, Y., and Liang, C. (2020). Drivers and outcomes of supply chain finance adoption: An empirical investigation in china. International Journal of Production Economics, 220:107453.
51. Wernerfelt, B. and Montgomery, C. A. (1988). Tobin’s q and the importance of focus in firm performance. The American Economic Review pages 246–250.
52. Wuttke, D. A., Blome, C., Foerstl, K., and Henke, M. (2013). Managing the innovation adoption of supply chain finance empirical evidence from six European case studies. Journal of Business Logistics, 34:148–166.
53. Wuttke, D. A., Rosenzweig, E. D., and Heese, H. S. (2019). An empirical analysis of supply chain finance adoption. Journal of Operations Management, 65:242–261.
54. Xu, X., Chen, X., Jia, F., Brown, S., Gong, Y., and Xu, Y. (2018). Supply chain finance: A systematic literature review and bibliometric analysis. International Journal of Production Economics, 204:160–173.
55. Zhao, X., Yeung, K., Huang, Q., and Song, X. (2015). Improving the predictability of business failure of supply chain finance clients by using external big datasets. Industrial Management & Data Systems
