# Supplementary material for: Light in the tunnel or just a train; impact of supply chain finance solutions on financial service providers’ financial performance by mitigating financial risk
Source: PLoS One. 2023 Dec 13;18(12):e0292497. doi: 10.1371/journal.pone.0292497 (PMC10718461; doi:10.1371/journal.pone.0292497)
Supplement: Attachment — Submitted filename: Response to Reviewers.docx [file pone.0292497.s004.docx]

**Author Rebuttal Letter**

**Dear Editor and reviewers**

First, I am great full for reviewing the research paper and for your valuable suggestions. All the suggestions and comments has been incorporated, which improve the document. The revised paper is being submitted with a request to consider this for publication.

**Following are the details with suggested changes have been incorporated**

**Reviewer 1:**

First, thank you for your time of reviewing the research paper with valuable suggestions. All the suggestions and comments has been incorporated, which improve the document. The revised paper is being submitted with a request to consider this for publication.

**Comments to the Author:** The author used dummy variables to denote the Supply Chain Finance (SCF) solutions offerings by Financial Service Providers (FSPs). If the FSP offer a particular SCF solution in a given year, then it is coded as 1, else 0. Thus, reducing the SCF solutions offering to binary variables. However, I have a main concern of using Principal component Analysis (PCA) for data reduction or the creation of SCF index. PCA is suitable for reducing the numeric multivariate variables. If it is a mixed of numeric variables and binary variables, PCA can still be useful. There is no justification on the appropriateness of the PCA for reducing binary variables. Further, the paper makes no comment on the number of SCF solutions offered by banks. What was the maximum number of SCF solutions offered by FSPs? It is not clear out of how many binary variables (SCF solutions), the SCF Index was created.

**Author Response:** Change has been incorporated into the document. The Principal Component Analysis technique of (Baker & Wurgler, 2007), is applied to quantify the supply chain finance solutions index using supply chain finance solutions as proxies. There is maximum 21 number of SCF solutions offered by FSPs.

**Comment to the Author:** “Using principal component analysis (PCA), the EViews index is created”- The writing needs to be clearer. What is views index? Do you mean SCF solution index is generated using PCA on views software?

**Author Response:** Change has been incorporated into the document. Supply chain finance solution index which is generated by using PCA on EViews software is correct statement.

**Comment to the Author:** For example, if a bank offers account receivables finance or an early payment discount program, it is coded as 1 and 0 if not offered buyer direct financing”. I have a concern with “0 if not buyer direct financing”. What does the author intend by this statement? Does the author mean that if the FSP is not offering any of the SCF solutions, then offering of buyer direct financing is assumed? If so then it could be wrong assumption. Just because a particular firm does not avail SCF solution from the FSP, does not mean it will always get buyer direct financing. A firm getting buyer direct financing may also simultaneously avail SCF solution from the bank.

**Author Response:** Change has been incorporated into the document. Each bank website check and mark how many supply chain finance solutions offered by particular bank out of list of the supply chain finance solutions, if a bank offers any one solution like account receivables finance or an early payment discount program it is coded as 1. On the other side if bank is not offering any particular supply chain finance solution it is coded as 0.

**Comment to the Author:** Table 2a: The Adjusted R2 keeps on declining when new variables are added (Models 3 and 4). It means the main variables SCF solution index and SCF x Financial Risk are not contributing to the overall model. How do you reconcile with the overall results?

**Author Response:** Table 2a financial performance measure with the proxy return on assets, the Adjusted R2 in table 2a keeps on declining the adjusted r-squared response for new variable and increase if new variable enhances the model above about what would be obtained by probability. On the other side it is decline as SCF x Financial Risk improves the model less than what is predicted. When new variable added in the model, adjusted R2 will automatically decrease.

**Comment to the Author:** “The coefficient is negative 0.0067, which means the moderating effect is interference rather than reinforcement. This suggests that the bank’s supply chain finance solution increases financial performance by increasing financial risk and supporting H2, which is the Supply chain finance solution that enables the financial performance of financial service providers to mitigate their financial risk”. The author states that the coefficient is negative 0.0067 whereas Table 2b shows it to be positive. The interpretation “This suggests that the bank’s supply chain finance solution increases financial performance by increasing financial risk and supporting H2, which is the Supply chain finance solution that enables the financial performance of financial service provider to mitigate their financial risk” is questionable.

**Author Response:** Change has been incorporated into the document. The coefficient is negative 0.0067, which means the moderating effect is interference rather than reinforcement. Financial risk reduces when financial service providers offer supply chain finance solutions with reinforcement of financial performance.

**Comment to the Author:** More care needs to be given in writing the overall manuscript.

**Author Response:** Change has been incorporated into the document. Prof-read all the document and made necessary changes in the manuscript.

**Reviewer 2**

First, thank you for your time of reviewing the research paper with valuable suggestions. All the suggestions and comments has been incorporated, which improve the document. The revised paper is being submitted with a request to consider this for publication.

**Comment to the Author:** English editing needed to make the document more readable. There are some paragraphs hard to read.

**Author Response:** Change has been incorporated into the document. Rewrite the paragraphs to make it more clear reading.

**Regards**

Authors
